# Effect of school reopening on SARS-CoV-2 incidence in a low-prevalence region: Prospective SARS-CoV-2 testing in healthcare workers with primary school-attending children versus without children living at home

**DOI:** 10.1177/17571774211012469

**Published:** 2021-06-18

**Authors:** Melvin Frie, Lisa M Havinga, Janneke Wiersema-Buist, Charlotte G Veldman, Marjan JT de Vries, Lilli Rurenga-Gard, Alex W Friedrich, Marjolein Knoester

**Affiliations:** 1 Department of Medical Microbiology and Infection Control, University of Groningen, University Medical Center Groningen, The Netherlands; 2 Department of Surgery, University of Groningen, University Medical Center Groningen, The Netherlands; 3 Department of Medical Sciences, University of Groningen, University Medical Center Groningen, The Netherlands; 4 Department of Occupational Health Service, University of Groningen, University Medical Center Groningen, The Netherlands

**Keywords:** Infection control, infection prevention, respiratory tract infection

## Abstract

Coronavirus disease 2019 (COVID-19) often presents asymptomatically or milder in children compared to adults. The role of young children in the transmission of severe acute respiratory syndrome coronavirus-2 (SARS-CoV-2) remains largely unknown. In the Netherlands, the first action of loosening the partial lockdown that had been implemented to reduce SARS-CoV-2 transmission was the reopening of primary schools on 1 May 2020. We subsequently conducted a prospective cohort study among healthcare workers (HCWs) with primary school-attending children versus HCWs without children living at home. We tested each HCW three times for SARS-CoV-2 from May 20 to June 15 2020 at 1-week intervals. In total, 832 nasopharyngeal swabs were taken from 283 HCWs with primary school-attending children living at home and 864 nasopharyngeal swabs from 285 HCWs without children living at home. All nasopharyngeal swabs tested negative for SARS-CoV-2. In our region with a low population density and low SARS-CoV-2 prevalence, reopening of primary schools did not lead to an increase in infections. The results of this study may serve as an example for the implementation of regional strategies to reduce SARS-CoV-2 transmission in countries with large variations in both population density and SARS-CoV-2 prevalence.

## Background

Coronavirus disease 2019 (COVID-19), the disease caused by severe acute respiratory syndrome coronavirus 2 (SARS-CoV-2), often presents asymptomatically or milder in children compared to adults (Dong et al, 2020; Guan et al, 2020). An analysis of SARS-CoV-2 viral load by patient age showed that age was not a predictor of SARS-CoV-2 viral load, and thus children may be as infectious as adults (Jones et al, 2020). However, it might be that the discrepancy is caused by the fact that children are often asymptomatic or too mildly infected to draw medical attention and thus be counted in the number of infected cases (Mehta et al, 2020; Viner et al, 2021). Information regarding the circulation of SARS-CoV-2 among children and the role of SARS-CoV-2 transmission from children to adults remains limited (Kelvin and Halperin, 2020).

On 11 May 2020, primary schools reopened in the Netherlands, as a first action of loosening up the partial lockdown that had been implemented in order to reduce SARS-CoV-2 transmission (Supplementary material) (Government of the Netherlands, 2020). To answer the question on potential transmission by children, the BackToSchool-study was initiated to investigate whether healthcare workers (HCWs) with primary school-attending children were more likely to become infected with SARS-CoV-2 compared to HCWs without children living at home.

This cohort study started after a period of active case finding among HCWs at the University Medical Center Groningen (UMCG). In the northern Netherlands, the first case of COVID-19 was diagnosed in the last week of February 2020 (National Institute for Public Health and the Environment, 2020). As of 10 March 2020, the UMCG actively tested all symptomatic UMCG-HCWs to prevent further transmission at work and within the community. We also present the results of this testing policy.

## Methods

The UMCG is the sole tertiary care centre in the northern part of the Netherlands supplying care for the provinces of Groningen, Friesland and Drenthe, a population of approximately 1.7 million inhabitants. As of 10 March 2020, HCWs of the UMCG were routinely tested by the occupational health service when showing symptoms compatible with COVID-19. If transmission within a department was likely, asymptomatic HCWs on the department were also tested. The number of HCWs tested and the numbers of positive and negative results were recorded.

The BackToSchool-study was a prospective cohort study among UMCG employees. A recruiting advertisement was posted in the daily digital newsletter. HCWs were eligible for inclusion if they were 18 years or older, had at least one primary school-attending child (study group) or had no children living at home (control group). An exclusion criterion was a previous positive test result for SARS-CoV-2 for the participant or their family members. Only one HCW per family could be enrolled. After reopening of primary schools on 11 May, from 20 May to 15 June 2020, participants were tested for SARS-CoV-2 by real-time polymerase chain reaction (RT-PCR) on nasopharyngeal and throat swabs (Supplementary material). Each participant was tested three times, at 1-week intervals. If symptoms compatible with COVID-19 occurred between two testing moments, an extra test was scheduled (Supplementary material). A baseline questionnaire was filled out prior to the first testing moment. An additional questionnaire regarding daily contacts, travel history and symptoms was filled out every testing day (Supplementary material).

To achieve 80% power with an α of 0.05, the minimum sample size per group was 270 including a 5% dropout.

This was based on the incidence of HCWs testing positive for SARS-CoV-2 at the time of design of this study (<1%) and the estimation of a difference between groups of 3%. Statistical analyses were performed using IBM SPSS 23.0.

## Results

**Figure 1.**
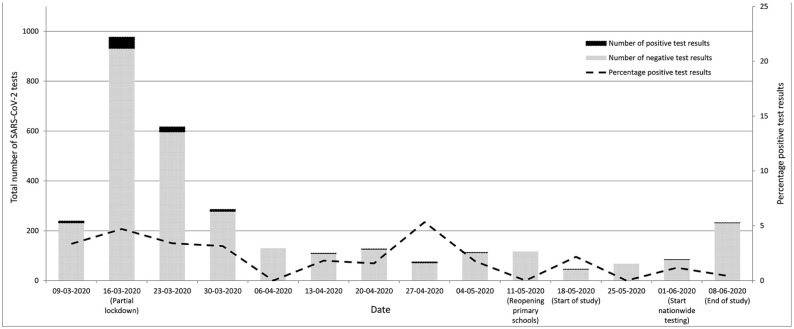
SARS-CoV-2 test result among symptomatic UMCG-healthcare workers at our centre prior to and during the BackToSchool-study (BackToSchool-study results not included).

Figure 1 shows the number of UMCG-HCWs tested per week, the number of positive and negative results and the test positivity rate from 10 March to 15 June 2020 (study samples not included). A peak in positive results was seen in March 2020, and declined afterwards. For the BackToSchool-study, 283 HCWs with primary school-attending children (mean age 42.1 years) and 285 HCWs without children living at home (mean age 45.7 years) were included. A total of 1696 nasopharyngeal swabs were taken (832 in the study group and 864 in the control group), and all tested negative for SARS-CoV-2. Thus, no difference in infection rates was detected between groups. Sociodemographic characteristics and questionnaire data are shown in Table 1.

**Table 1.** Characteristics and questionnaire data of the study population.

**Variable**	**Study group**	**Control group**
	HCWs with primary school-attending children (*n*=283)	HCWs without children living at home (*n*=285)
Age, mean (SD), in years	42.1 (5.4)	45.7 (14.5)
Sex		
Male	60 (21.2%)	58 (20.4%)
Female	223 (78.8%)	227 (79.6%)
Total no. of SARS-CoV-2 test results		
Positive	0 (0%)	0 (0%)
Negative	837 (100%)	864 (100%)
Total no. of nasopharyngeal swabs taken	832	864
Total no. of faecal testing	5	0
Inconclusive test result and retesting required	15	29
No. of participants who completed 3 or more testing moments	263 (92.9%)	273 (95.8%)
No. of participants who completed 2 testing moments	14 (4.9%)	11 (3.9%)
No. of participants who completed 1 testing moment	6 (2.1%)	1 (0.4%)
BMI, median (IQR)	23.8 (21.8-26.1)	23.9 (21.8-26.5)
Education level^a^		
Low	10 (3.5%)	18 (6.3%)
Middle	33 (11.7%)	34 (11.9%)
High	240 (84.8%)	233 (81.8%)
Type of work		
Direct contact with patients	145 (51.2%)	132 (46.3%)
No direct contact with patients	12 (4.2%)	4 (1.4%)
No contact with patients or their environment	126 (44.5%)	149 (52.3%)
Contact with children at work		
Yes	21 (7.4%)	22 (7.7%)
No	262 (92.6%)	263 (92.3%)
Family status		
Partnered	261 (92.2%)	189 (66.3%)
Single	22 (7.8%)	96 (33.7%)
Partner’s type of work		
HCW with contact with children	15 (5.7%)	7 (3.7%)
HCW without contact with children	54 (20.7%)	26 (13.8%)
No HCW, but contact with children	7 (2.7%)	8 (4.2%)
A contact-based profession	14 (5.4%)	7 (3.7%)
Driver instructor or bus driver	1 (0.4%)	0 (0%)
Other than mentioned above	170 (65.1%)	141 (74.6%)
Household region^b^		
An urban or suburban area within the three northern provinces	144 (50.9%)	181 (63.5%)
An urban or suburban area outside the three northern provinces	1 (0.4%)	0 (0%)
A rural area within the three northern provinces	138 (48.8%)	102 (35.8%)
A rural area outside the three northern provinces	0 (0%)	2 (0.7%)
Family size, no. of members, median (IQR) [range]	4.0 (4.0-5.0), [2.0-8.0]	2.0 (1.0-2.0), [0.0-7.0]
No. of children living at home aged <18 years		
<2	42 (14.8%)	NA
2	159 (56.2%)	NA
>2	81 (28.6%)	NA
Unknown	1 (0.4%)	NA
No. of children living at home attending primary school		
<2	156 (55.1%)	NA
2	101 (35.7%)	NA
>2	25 (8.8%)	NA
Unknown	1 (0.4%)	NA
**Social contacts, travel history and exposure to ill persons**		
No. of contacts outside the working environment and family household 7 days prior to a testing moment, median (IQR)^c^	3.0 (1.0-6.0)	3.0 (1.0-6.0)
No. of participants with ⩾ 1 contacts with a person living or working outside the three northern provinces 7 days prior to a testing moment^b,c^	113 (13.5%)	146 (16.9%)
No. of participants with ⩾ 1 travel movements outside the three northern provinces 7 days prior to a testing moment^b^	82 (9.8%)	135 (15.6%)
Direct contact without preventive measures with a person tested positive for SARS-CoV-2 the last 14 days prior to a testing moment^c^		
Yes	4 (0.5%)	1 (0.1%)
No	812 (97.0%)	851 (98.5%)
Unknown	21 (2.5%)	12 (1.4%)
Coronavirus-like symptoms within the family 7 days prior to a testing moment^d^		
Yes	73 (8.7%)	17 (2.0%)
No	743 (88.8%)	834 (96.5%)
Unknown	21 (2.5%)	13 (1.5%)

a Education categories were defined as: low = high school graduate or lower; middle = college education but no college degree; high = college degree or higher.

b The three northern provinces of the Netherlands include the provinces of Friesland, Groningen, and Drenthe.

c Defined as having contact with others for 15 minutes or longer, at a distance less than 1.5 metres, without wearing protective facial mask, glasses or comparable protective clothing.

d Coronavirus-like symptoms included symptoms of fever, shortness of breath, muscle ache, (dry) cough, sore throat, runny nose, fatigue, loss of taste or smell, headache or (unexplained) diarrhoea. HCWs: healthcare workers; SD: standard deviation; IQR: interquartile range; SARS-CoV-2: severe acute respiratory syndrome coronavirus 2; NA: not applicable.

## Discussion

After reopening primary schools, we found no increased SARS-CoV-2 incidence among HCWs compared to previous weeks. Nor did we find a difference in SARS-CoV-2 incidences between HCWs with primary school-attending children versus HCWs without children living at home. In fact, no infections were detected at all. To put these findings in perspective, the epidemic in the Netherlands evolved from the beginning of March, peaked in April and stabilised at low frequency in May and June (Supplementary Figure 1). The epidemic started in the south of the Netherlands and before it had reached the northern provinces, the partial lockdown was introduced country-wide.

Despite the early implementation of the partial lockdown in our region, infections did occur (Supplementary Figure 2). However, the cumulative prevalence in our region until July 21 2020 was 91/100,000 inhabitants, compared to the Dutch total of 299/100,000 inhabitants (National Institute for Public Health and the Environment, 2020).

Nationwide screening of all symptomatic persons was introduced in the Netherlands on 1 June 2020, with a nationwide positivity rate during the BackToSchool-study of 1.6% (1880/116,764) and of 0.5% (41/7703) for the three northern provinces (National Institute for Public Health and the Environment, 2020). We did not expect the incidence to drop so low that comparison between study groups would be hampered. Postponing the study to a later moment in time, e.g. in autumn or during a regional outbreak, might have increased our statistical power as a result of a higher background incidence. However, the moment of opportunity of only schools being reopened after a period of partial lockdown made us decide not to postpone. Antibody testing prior to the study was not performed as we believe that only a very small percentage of the HCWs included in this study will have unknowingly been infected, due to the active testing strategy in the preceding months and the low seroprevalence in our region (Slot et al, 2020).

The majority of positive cases in the UMCG were UMCG-HCWs (69%). By very early and active testing of all symptomatic HCWs, and excluding those with a positive test from working, we were able to reduce transmission of SARS-CoV-2 in our hospital. This service was promptly extended to all HCWs in critical professions in the provinces of Groningen and Drenthe, in cooperation with the Municipal Health Services and regional laboratories. In this collaboration, we also offered testing to symptomatic family members of HCWs, before the nationwide screening was initiated. This contributed amongst many other factors to a very low reproductive number in the northern Netherlands.

A cross-sectional study conducted in the southern province of Noord-Brabant showed that 6% out of 1353 symptomatic HCWs tested positive for SARS-CoV-2 and that the majority only experienced mild symptoms (Kluytmans-van den Bergh et al, 2020). It is of importance to actively test HCWs for SARS-CoV-2 even if only very mild symptoms are being reported and even more so when policies allow HCWs to work with mild symptoms. Furthermore, testing pre-/asymptomatic HCWs after being exposed to a COVID-19-infected person is crucial in a preventive search-and-contain policy within healthcare institutions.

The findings of this study suggest that reopening primary schools in areas with a low population density and low SARS-CoV-2 incidences will not cause disproportional SARS-CoV-2 transmission in this area. However, it is important to state that our study does not exclude that in another epidemiological context, with a higher incidence, introduction of positive cases into schools could have led to enhanced transmission of SARS-CoV-2.

## Supplemental Material

sj-docx-1-bji-10.1177_17571774211012469 – Supplemental material for Effect of school reopening on SARS-CoV-2 incidence in a low-prevalence region: Prospective SARS-CoV-2 testing in healthcare workers with primary school-attending children versus without children living at home Click here for additional data file. Supplemental material, sj-docx-1-bji-10.1177_17571774211012469 for Effect of school reopening on SARS-CoV-2 incidence in a low-prevalence region: Prospective SARS-CoV-2 testing in healthcare workers with primary school-attending children versus without children living at home by Melvin Frie, Lisa M Havinga, Janneke Wiersema-Buist, Charlotte G Veldman, Marjan JT de Vries, Lilli Rurenga-Gard, Alex W Friedrich and Marjolein Knoester in Journal of Infection Prevention
