# Supplementary material for: Effect of school reopening on SARS-CoV-2 incidence in a low-prevalence region: Prospective SARS-CoV-2 testing in healthcare workers with primary school-attending children versus without children living at home
Source: J Infect Prev. 2021 Jun 18;22(6):269–74. doi: 10.1177/17571774211012469 (PMC8647639; doi:10.1177/17571774211012469)
Supplement: sj-docx-1-bji-10.1177_17571774211012469 – Supplemental material for Effect of school reopening on SARS-CoV-2 incidence in a low-prevalence region: Prospective SARS-CoV-2 testing in healthcare workers with primary school-attending children versus without children living at home [file sj-docx-1-bji-10.1177_17571774211012469.docx]

**Supplementary Online Content**

**Timeline of loosening preventive measures in the Netherlands**

In late February, 2020, the first positive cases of severe acute respiratory syndrome coronavirus 2 (SARS-CoV-2) were confirmed in the Netherlands. Approximately two weeks later, on March 12, 2020, the Dutch government announced that meetings with more than 100 people were forbidden. However, schools remained open. On March 15, 2020, public measures were tightened up, schools and childcare providers were closed, all restaurants, cafés, sport clubs and saunas were closed, everyone was advised to stay at home, work from home if possible and to avoid public transport; a so-called intelligent/partial lockdown. After infection rates, hospital admissions and deaths due to SARS-CoV-2 declined, preventive measures were loosened step by step (*Government of the Netherlands., 2020*);

Relaxation of rules prior to or during the BackToSchool-study:

As of May 11, 2020:

Primary schools (children aged 4-12 years) and childcare providers (children aged 0-12 years) reopened, with the restriction that only 50% of children were allowed to be present.

Contact-based professionals were allowed to resume their work. This included dentists, hairdressers, physical therapists, dieticians, driving instructors, etc.

Outdoor activities including sports were again allowed if people keep 1.5 metres of distance. However, this distance rule did not apply to children under the age of 12.

As of June 2, 2020:

Secondary schools (age 12-18) reopened, with scholars keeping 1.5 metres distance.

Buildings open to the public could admit up to 30 people at one time.

Meeting people outside was again allowed, keeping 1.5 metres of distance.

Restaurants, bars and cafés reopened under the following conditions: a maximum of 30 guests at one time, reservation obliged and everyone must keep 1.5 metres distance, except people from the same household.

All passengers aged 13 years or older must wear a facial mask in public transport.

As of June 8, 2020:

Primary schools open with regular hours and full classes.

**Epidemiological situation of SARS-CoV-2 in the Netherlands prior to and during the BackToSchool-study**

The epidemic started in the south of the Netherlands in the province of Noord-Brabant. During the epidemic, the local Municipal Health Services were responsible for visualizing the current situation and contact tracing. Data were retrieved by the government on a daily basis and made available for the public. **Figure 1** shows the national number of confirmed SARS-CoV-2 cases as of the start of the epidemic in the Netherlands (*National Institute for Public Health and the Environment., 2020*). **Figure 2** shows the number of notified SARS-CoV-2 cases per 100.000 in habitants per province at the peak of the epidemic and during the BackToSchool-study (*National Institute for Public Health and the Environment., 2020*).

Figure 1. Number of confirmed SARS-CoV-2 cases from the start of the epidemic in the Netherlands.


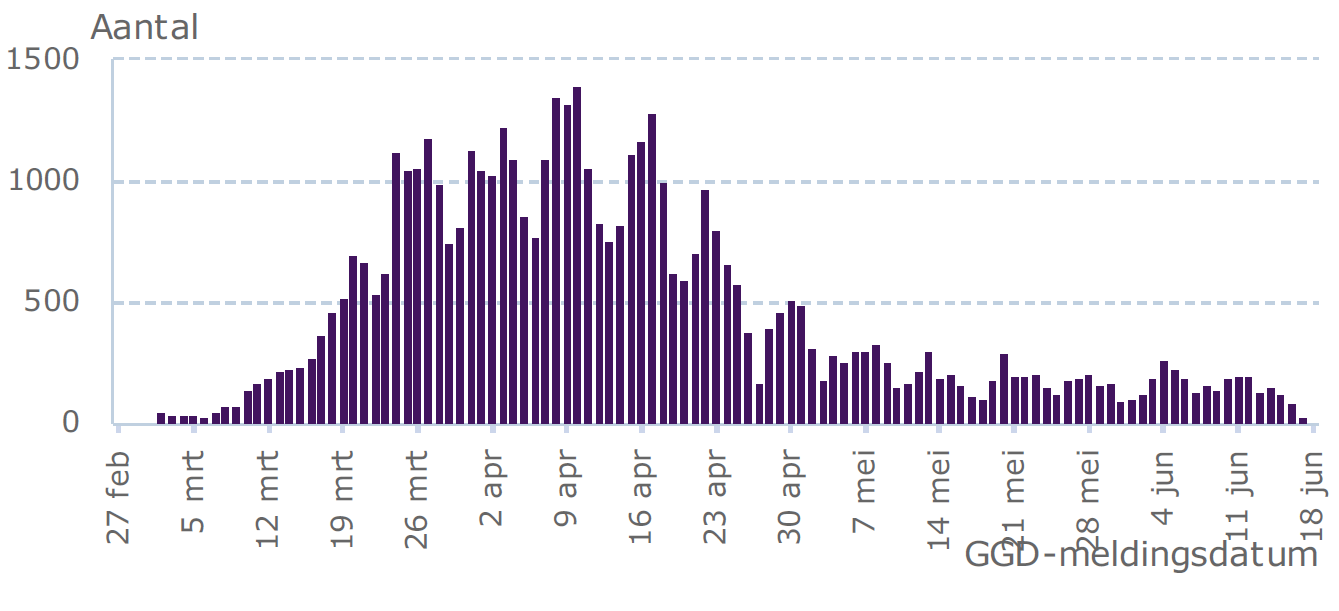


**Figure 2.** Reported COVID-19 patients per 100.000 inhabitants per county at the peak of the epidemic (left) and during the BackToSchool-study (right).


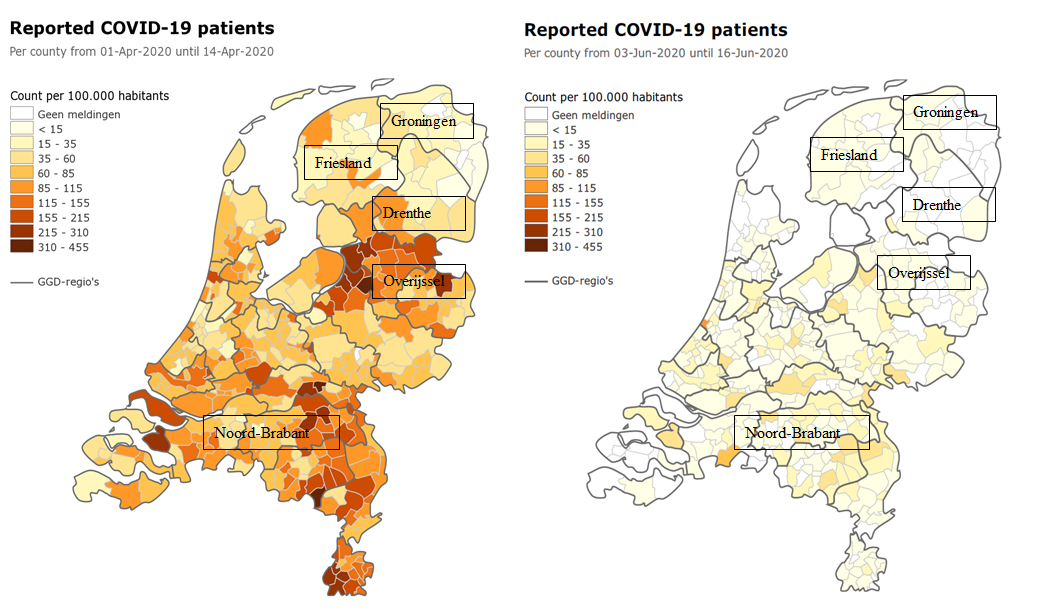


**RT-PCR protocol**

Before extraction, stool samples were suspended in NucliSens easyMAG lysisbuffer (2×volume of feces, bioMérieux, Marcy l’Etoil, France). After centrifugation, 100 µl of the supernatant was used for the extraction and from the nasopharyngeal swabs 190 µl. The samples were extracted to the manufacturer’s instructions, with the addition of 10 µl internal control, phocine distemper virus (PDV), using the NucliSense EasyMag (Biomerieux, Lyon, France). SARS-CoV-2 RT-PCR was performed on the E-gen as described by Corman et al. with minor modifications (*Corman et al., 2020*). The internal control PCR reaction is a multiplex with SARS-CoV-2 and is performed in a total reaction volume of 25µl using 10µl RNA and 15µl PCR mix, containing 1xTaqMan® Fast Virus 1-Step Master (Applied Biosystems, Foster City, CA, USA), DNAse/RNAse free water (Sigma, The Netherlands), 400nM SARS-CoV-2 forward and reverse primer, 200nM SARS-CoV-2 probe, 300nM PDV forward primer (5’-cgggtgccttttacaagaac), 300nM PDV reverse primer (5’-ttctttcctcaacctcgtcc) and100nM PDV probe (NED-aag ggc caa ttc t-MGBNFQ). The ABI PRISM 7500 (Life technologies, USA) was used for the amplification and detection using the profile of 2 min 50°C, 20 s 95°C, followed by 45 cycles of 3 s 95°C and 32 s 60°C.

**Additional testing**

Participants were requested to contact the study team if they experienced any symptoms of fever, shortness of breath, muscle pain, (dry) cough, sore throat, nose cold, fatigue, pain behind the eyes, loss of taste or smell, headache or (unexplained) diarrhea. Participants who reported any gastrointestinal symptoms, feces was also tested for SARS-CoV-2 according to the same protocol as nasopharyngeal swabs.

**Questionnaire filled out at every testing day**

1. Today is my …… testing moment.

1st

2nd

3rd

2. How many people have you had contact with in the past 7 days? Please fill in the number of people (for example 0, 3 or 7).

*Explanation: this includes all contacts outside of your own household and work. By ‘contact’ we mean at a distance less than 1.5 metres to another person, without protective, preventive measures (mask, gloves, protective clothing). Casual encounters in for example supermarkets should not be included.*

3. How many times have you been outside the three Northern provinces (Groningen, Friesland, and Drenthe) in the past 7 days? Fill in the number of times (for example 0, 3 or 7). If you live outside of these provinces, please fill in ‘100’.

4. How many times have you been outside of the four Northern provinces (Groningen, Friesland, Drenthe, and Overijssel) in the past 7 days? Fill in the number of times (for example 0, 3, or 7) If you live outside these provinces, please fill in ‘100’.

5. Has anyone in your household (including yourself) had any new symptoms that may be consistent with the new coronavirus in the past 7 days?

*Explanation: Symptoms that may be consistent with the new coronavirus include: body temperature above 37.5 degrees Celsius (when using paracetamol/aspirin you must add 0.5 degrees to the temperature measured), shortness of breath, muscle pain, sore throat, nose cold, loss of smell or taste, extreme fatigue, headache and/or pain behind the eyes or (unexplained) diarrhoea.*

No

Yes

6. In the past 14 days, have you had close contact* with someone who has tested positive for the new coronavirus?
*If you have contact with patients with corona professionally, you do not need to include these contacts because of preventive measures taken.*
**On a distance less than 1.5 metres, without protective, preventive measures, for 15 minutes or longer.*

No

Yes

7. How many times in the past 7 days have you had close contact* with someone who lives or works outside of Groningen, Friesland, Drenthe or Overijssel? Only fill in the number of times (for example 0, 3 or 7). If your partner lives or works outside of these four provinces, please fill in ‘100’.
**On a distance less than 1.5 metres, without protective, preventive measures, for 15 minutes or longer.*

**References**

Corman, V.M., Landt, O., Kaiser, M., Molenkamp, R., Meijer, A., Chu, D.K., Bleicker, T., Brunink, S., Schneider, J., Schmidt, M.L., Mulders, D.G., Haagmans, B.L., van der Veer, B., van den Brink, S., Wijsman, L., Goderski, G., Romette, J.L., Ellis, J., Zambon, M., Peiris, M., Goossens, H., Reusken, C., Koopmans, M.P. & Drosten, C. 2020, "Detection of 2019 novel coronavirus (2019-nCoV) by real-time RT-PCR", *Euro surveillance : bulletin Europeen sur les maladies transmissibles = European communicable disease bulletin,*vol. 25, no. 3, pp. 10.2807/1560-7917.ES.2020.25.3.2000045.

Government of the Netherlands 2020, *, Changes to the coronavirus control measures from 11 May 2020*. Available: [https://www.government.nl/topics/coronavirus-covid-19/tackling-new-coronavirus-in-the-netherlands/changes-to-measures-from-11-may](about:blank) [2020, May 29,].

National Institute for Public Health and the Environment 2020, *, Current information about COVID-19. COVID-19 in the Netherlands in graphs*. Available: <https://www.rivm.nl/en/novel-coronavirus-covid-19/current-information> [2020, June 19,].
